# Core Circadian Protein BMAL1: Implication for Nervous System Functioning and Its Diseases

**DOI:** 10.3390/brainsci15121321

**Published:** 2025-12-11

**Authors:** Kristina V. Smirnova, Liudmila P. Smirnova, Tamara G. Amstislavskaya

**Affiliations:** 1 Research Institute of Mental Health, Tomsk National Research Medical Center, Russian Academy of Sciences, Aleutskaja, 4, 634014 Tomsk, Russia; 2 Scientific Research Institute of Neurosciences and Medicine, Timakova Street, 4, 630117 Novosibirsk, Russia

**Keywords:** BMAL1, circadian rhythm, neuroinflammation, neurodegeneration, neurodevelopment, cognitive function, mood disorders

## Abstract

The brain and muscle ARNT-like 1 protein, also known as BMAL1 or ARNTL1, is one of the key transcriptional regulators of circadian rhythms that controls the diurnal dynamics of a wide range of behavioral, hormonal, and biochemical factors in most living creatures around the Earth. This protein also regulates many physiological processes, and its disruption leads to pathological conditions in organisms, including nervous system disorders. The high evolutionary conservativity of BMAL1 allows for the construction of in vitro and in vivo models using experimental animals and the investigation of BMAL1-dependent molecular mechanisms of these diseases. In this review, we have collected data from human and animal studies concerning the roles of BMAL1 in processes such as neuroinflammation, trauma and neurodegeneration, neurodevelopment and myelinization, mood disorders, addictions, cognitive functions, and neurosignaling. Additionally, we provide information about the biochemical regulation of BMAL1 and pharmacological approaches to change its activity. Here, we conclude that BMAL1 functions in the nervous system go far beyond circadian rhythm regulation in most cell types, including neurons, glial cells, and microglial cells. Under pathological conditions, lack or overexpression of this protein can exert both protective and destructive effects. Thus, proper therapeutic modulation of BMAL1 activity is a promising approach for improving nervous system disorders.

## 1. Introduction

The BMAL1 (brain and muscle ARNT-like 1) protein is one of the key circadian transcription factors in living creatures around the Earth, which controls the diurnal rhythm of gene expression and physiological processes such as metabolism, immunity, and redox homeostasis, and is also involved in aging [1,2]. Growing scientific evidence shows that BMAL1 is an important factor for neuroprotection, and its disruption is involved in the development of several nervous system diseases [3].

**Figure 1:**
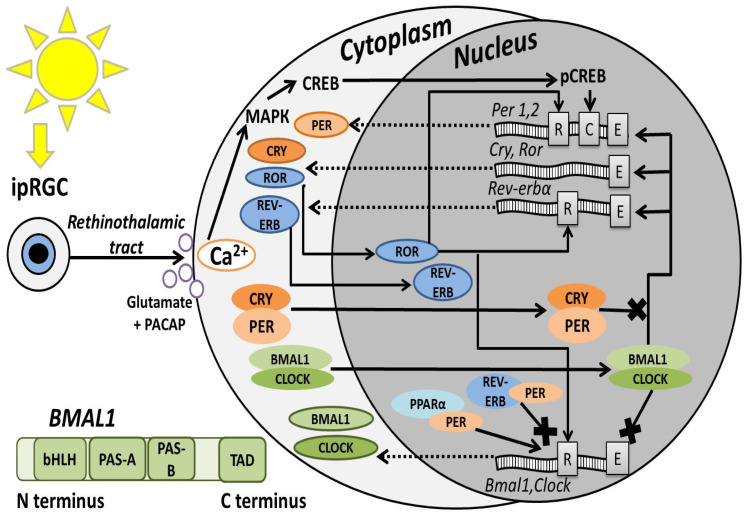
Circadian oscillation scheme based on the literature data presented in the chapter “BMAL1 molecular regulation”. Black arrows indicate activating actions, arrows with a cross—inhibition, and dotted arrows indicate nucleus-to-cytoplasm transport of the mRNAs. Gray rectangles on the DNA indicate certain promoter regions: R—RRE or REV responsible element; C—CRE or cAMP regulatory element; E—E-box. Green rectangle in the lower part—schematic BMAL1 protein structure with functional domains based on Gustafson et al., 2017 [6].

BMAL1 is a member of the transcription factor family that contains bHLH (basic helix-loop-helix) and PAS (Per-ARNT-Sim) domains. These factors control gene expression and affect a wide range of functions, including the response to external influences, hypoxia, neurogenesis, synaptic plasticity, and, more typically for BMAL1, circadian rhythm [4]. Genes containing bHLH/PAS domains are evolutionarily conserved, and their functional divergence took place before vertebrates. There are three groups of bHLH/PAS-containing genes: the first includes AHR (aryl hydrocarbon receptor); the second includes SIM1/2 (single-minded homolog), HIF-1-3α (hypoxia inducible factor), and NPAS (neuronal PAS domain) 1/3; and the third group includes two large clades of genes: the NPAS2/CLOCK (circadian locomotor output cycle kaput) clade and the second, which is divided into two subclades, ARNT/ARNT2 (aryl hydrocarbon receptor nuclear translocator) and ARNTL/ARNTL2 (also known as BMAL1/2) [5]. The BMAL1 protein contains bHLH and two PAS (A and B) domains. The latter is critically important for recognition and interaction with specialized DNA regions on promotors called E-box sequences and plays a role in dimerization with proteins, such as CLOCK [4]. The BMAL1 C-terminus contains a transactivation domain (TAD) where interactions with transcriptional coactivators or corepressors occur (Figure 1, lower part) [6].

The high level of evolutionary conservativity of BMAL1 in different vertebrate species allows for the creation of valid animal models and the study of BMAL1 molecular functions in physiological and pathological conditions, including human nervous system diseases (Table A1) [1,7]. This review summarizes data on BMAL1 functions in different cell types of the mammalian nervous system and its implications for brain pathologies, including trauma, neurodegeneration, and mood disorders. Additionally, data concerning BMAL1 molecular regulation and the therapeutic effect of exposure on protein activity are presented.

## 2. BMAL1 Molecular Regulation

First, it is necessary to discuss the role of BMAL1 in the circadian rhythm because this protein has a key role in these processes and is simultaneously regulated by other molecular players of diurnal oscillations (Figure 1). The mammalian circadian system has a hierarchical structure: the master clock is located in the brain and is presented as a paired hypothalamic suprachiasmatic nucleus (SCN), which controls the biorhythm of all tissues and cells in an organism, defines the time of main physiological events (digestion, the sleep-wake cycle, motor activity, etc.) [8]. In mammals, light entrains circadian gene oscillations via melanopsin-expressing intrinsically photosensitive retinal ganglion cells (ipRGC), which project to the SCN through the retinohypothalamic tract, where neurotransmission relies on glutamate and pituitary adenylate cyclase-activating polypeptide (PACAP). As a result, in the SCN ventromedial zone, neurons increase the intracellular Ca^2+^ concentration, which activates the mitogen-activated protein kinase (MAPK) signaling cascade and cAMP response element-binding protein (CREB) phosphorylation; after that, pCREB translocates into the nucleus, where it binds to the CRE element on the *Period* (*Per*) promoter [9,10,11]. The light induction of these genes is time-limited but sufficient to trigger subsequent events [12].

The CLOCK (or its paralog NPAS2) and BMAL1 proteins accumulate in the cytoplasm during the dark phase (in diurnal animals), form a complex, enter the nucleus, and bind to the E-box elements on various gene promoters, including circadian ones, activating their transcription. Finally, PER and CRY (cryptochrome) proteins accumulate in the cytoplasm, dimerize, translocate to the nucleus, and attach to the CLOCK:BMAL1 complex, thereby disrupting its interaction with gene promoters and inhibiting transcriptional activity. This in consequence decreases *Per* and *Cry* transcription, forming a self-sustaining negative feedback loop that oscillates with a cycle of approximately 24 h [13,14]. Several transcription factors regulate the expression of *Bmal1* by binding to the RRE promoter region of this gene: the REV-ERBα protein (also known as NR1D1 nuclear receptor subfamily 1 group D member 1) inhibits its transcription, and the RORα protein (tyrosine kinase-like orphan receptor) activates it [15]. Moreover, the CLOCK:BMAL1 complex binding to the E-box sequence of *Bmal1* leads to the inhibition of *Bmal1* transcription, whereas the PER:CRY complex has a positive effect on it [16]. PER2 is also able to interact with the nuclear receptors REV-ERBα and PPARα (peroxisome proliferator-activated receptor α) on *Bmal1* promoters, which act as transcriptional corepressors and coactivators, respectively [17].

In addition to the main circadian mechanism of BMAL1 regulation, many other factors affect the gene expression or activity of this protein (Table 1). For example, the methylation of CpG sites in the human *BMAL1* gene promoter represses its activity [18]. Transcription factors such as DEC1/2 (deleted in esophageal cancer1/2), NF-κB (nuclear factor kappa-light-chain-enhancer of activated B cells)-p65, and DCNP1 (dendritic cell nuclear protein 1) interact with BMAL1 or compete with it for E-box binding to sequences on gene promoters [19,20,21]. MicroRNAs (miRs) repress *Bmal1* mRNA translation by binding to a specific 3′ untranslated region [22,23]. Additionally, human *BMAL1* mRNA undergoes alternative splicing, leading to the appearance of two protein isoforms with different properties [24]. TADs at the BMAL1 C-terminus can isomerize over time, which affects rhythm stability [6]. Also, BMAL1 activity is affected by a number of post-translational modifications through interactions with glycogen synthase kinase-3 (GSK-3) [25,26], calmodulin-dependent protein kinase (CaMK) IIδ [27], and some kinases in the mammalian target of rapamycin (mTOR) pathway [28,29], as well as through interactions with NAD-dependent histone deacetylase silent information regulator 1 (SIRT1) [30,31,32].

Among other factors, the activity of BMAL1 in peripheral tissues and in the SCN has specific characteristics, since peripheral tissue cells differ in terms of chromatin availability and E-box sequence structure [33,34,35]. Moreover, *Bmal1* gene expression is different in various cells. Thus, in the mouse central nervous system (CNS), the highest *Bmal1* gene expression is shown in Purkinje cells, parvalbumin-positive prefrontal cortex (PFC) neurons, somatostatin-positive interneurons, and astrocytes. In some brain areas, such as the posterior cortex and striatum, increased *Bmal1* expression has been observed in inhibitory neurons, but in the hippocampus, *Bmal1* expression is increased in excitatory neurons and astrocytes [37]. Importantly, phases and peaks of circadian gene expression in peripheral tissues, but not the SCN, differ in phase by approximately 12 h between diurnal and nocturnal animals, which should be taken into account when planning experiments. For example, *Bmal1* expression in diurnal primates (*Papio anubis*) peaks in the evening; on the contrary, in mice (*Mus musculus*), which are predominantly nocturnal animals [38]. In simpler model organisms, the circadian system and circadian genes are structured differently than those in mammals. For example, Danio rerio fish do not have central pacemaker circadian rhythms and have 2 copies of bmal1 genes (bmal1a and b), although these copies have a similar circadian phase [39].

## 3. Physiological BMAL1 Functions in the CNS

### 3.1. BMAL1 in Inflammation, Oxidative Stress, and Neuroprotection Processes

BMAL1 is involved in various functions beyond circadian rhythm regulation throughout all organisms, including the CNS [3]. One of the most important BMAL1 functions is regulating inflammatory and oxidative stress response; for example, BMAL1 is a crucial antioxidant in myeloid cells, and its removal disrupts NRF2 (nuclear factor erythroid 2-related factor 2) activity, promoting the accumulation of reactive oxygen species (ROS) and the proinflammatory interleukin (IL)-1β [40]. In neurons, the BMAL1-NRF2 pathway also increases resistance to oxidative stress [41]. In isolated neuronal precursor cells (NPC), BMAL1 regulates the expression of genes involved in ROS detoxification, including *Catalase*, and enzymes involved in the reduction of reactive quinones and oxidized proteins, such as *Aldh2* (aldehyde dehydrogenase 2) and *Nqo1* (NAD(P)H quinone dehydrogenase 1) [41,42]. However, BMAL1 may have a differentiated effect on proinflammatory cytokines because BMAL1 deficiency decreases basal *Nf-κb* but increases *Il-6* mRNA expression [43].

Microglia in the CNS have many important immune functions, such as antigen presentation, cytokine secretion, and phagocytosis. Their activation during neuroinflammation leads to the release of proinflammatory agents and the stimulation of ROS generation [44]. BMAL1 has an ambiguous effect on the functions of these macrophages. *Bmal1* gene knockout (KO) in microglial cell culture reduces the expression of proinflammatory genes (*Il-1b* and *Nox2*) and increases the expression of anti-inflammatory and antioxidant genes (*Gsr* and *Hmox1*), thereby reducing inflammatory responses and simultaneously enhancing glucose intake and phagocytosis without increasing ROS activity [45]. Whereas in microglial cell cultures stimulated with lipopolysaccharide (LPS), the phosphorylation of NF-κB inflammatory pathway proteins (IκBα and p65) was additionally enhanced in the group with *Bmal1* silencing (si-Bmal1), which was accompanied by an increase in ROS production [46]. Another study of LPS-stimulated cultures of microglia revealed similar results: an increase in microglial phagocytic activity and the expression of proinflammatory IL-6, TNF-α (tumor necrosis factor-α), and BMAL1, whereas si-Bmal1 in these cells reversed changes in microglia. Interestingly, BMAL1 suppression in healthy cells has an effect similar to LPS stimulation [47]. This effect may be explained by the ability of microglia to shift their functional activity to maintain tissue homeostasis. In response to proinflammatory factors, macrophages can have an inflammatory M1 phenotype, or in response to anti-inflammatory factors such as IL-4 and IL-13, they can develop an M2 phenotype to reduce inflammation and repair tissue. The metabolic reprogramming of microglial cells plays a key role in this process [48]. Since BMAL1 regulates metabolic processes, it may therefore play a role in determining the phenotype of these cells under various conditions [45].

Brain protection is also carried out by the blood–brain barrier (BBB), where BMAL1 controls permeability and integrity via the regulation of platelet-derived growth factor receptor β (PDGFRß) in pericytes, which is necessary for maintaining the BBB [49]. Astrocytes can also control BBB permeability; regulate the extracellular ion concentration, neuronal lactate and glucose metabolism, and immune responses; and promote brain recovery after injury, which is necessary for CNS protection [50]. In astrocytes, BMAL1 is partially involved in glutathione-S-transferase signaling, suppresses cell-autonomous astrocyte activation (i.e., it is not associated with increased cell division), and the expression of inflammatory genes [51]. Additionally, BMAL1 plays a part in processes of astrocyte autophagy: deficiency of this protein increases endocytosis, lysosomal protein degradation, and the accumulation of LAMP1 (lysosome membrane protein 1)- and RAB7 (late endosome marker)-positive organelles via a TFEB (transcription factor EB)-independent mechanism [52].

Thus, BMAL1 promotes CNS protection through multiple mechanisms: it regulates inflammatory processes and oxidative stress in various cells, maintains blood–brain barrier (BBB) integrity, suppresses astrocyte activation, and modulates autophagy.

### 3.2. BMAL1 in Neurodevelopment and Myelinization

BMAL1 is involved in CNS development processes. During the perinatal period in mice, *Bmal1* gene transcripts accumulate in the cerebral cortex, peaking on the 3rd day after birth, promoting radial neuronal migration in the embryonic cortex and proliferation of axonal projections [53]. Also, BMAL1 is important for adult neurogenesis. In mammals, these processes occur mainly in two areas: the subventricular zone of the lateral ventricle (SVZ) and the dentate gyrus (DG) of the hippocampus. In the DG, adult neurogenesis includes proliferation, migration, and differentiation/maturation of NPCs and their integration into hippocampal circuits. Experience-dependent neurogenesis can lead to network formation in the DG, which is suitable for encoding new memories in familiar contexts and has an obvious connection with spatial memory formation and cognition [42,54]. The reduction in BMAL1 in DG granule cells subsequently reduces BMAL1 levels in neighboring neuronal stem cells in the SVZ, increasing their proliferation [55]. Moreover, BMAL1-KO mice present a decrease in the hippocampal NPC pool with a higher survival rate and scattered distribution but more frequent differentiation into astroglial cells that accelerate aging of the hippocampal neurogenic niche, accompanied by increased expression of SIRT1, PMP70 (70 kDa peroxisomal membrane protein), Waf1/CIP1 (cyclin-dependent kinase inhibitor 1), and oxidative stress [56]. Cortical-specific BMAL1-KO disrupts the proteome composition and its rhythmicity in the neocortex and hippocampus by increasing the expression of astrocyte marker proteins (GFAP and FABP7) and the expression of the enzyme ENTPD2 (ectonucleoside triphosphate diphosphohydrolase 2), which is involved in extracellular signaling during neurogenesis. Thus, BMAL1 is also necessary for the proper cellular composition of the anatomical structures of the brain [57]. On the other hand, BMAL1-KO in forebrain neurons reduces proliferation but accelerates NPC migration in the rostral migration stream from the SVZ to the olfactory bulb, which is accompanied by oxidative stress in subregions of the hippocampus and olfactory bulb but does not affect neurogenesis in these mice and does not lead to astrogliosis, indicating a region-specific BMAL1 role in CNS development [42].

BMAL1 suppression in the SVZ stimulates the transformation of NPCs into oligodendrocyte precursor cells (OPC) [58]. The main function of oligodendrocytes (OL) is the formation of myelin sheaths on nerve fibers. BMAL1 has an ambiguous role in OPC maturation, myelination, and remyelination processes. BMAL1-KO specific to OPCs during development disrupts the expression of genes related to the proliferation, density, morphology, and migration of these cells, as well as to the thinning of myelin [59]. On the other hand, the overexpression of BMAL1 in OPCs also negatively affects myelinization via the inhibition of the AKT/mTOR pathway and a reduction in OL differentiation gene expression [60]. Moreover, circadian-dependent suppression of BMAL1 synthesis via the Wnt pathway inhibitors SFRP1 and SFRP5 (secreted frizzled-related protein) produced by astrocytes stimulates oligodendrogenesis in the SVZ [58].

In summary, BMAL1 is an important factor determining CNS cell populations, influencing precursor cell survival and migration through transcriptional control. Importantly, these regulatory functions are highly context-dependent, varying by cell type and anatomical location—a critical consideration for experimental design.

### 3.3. BMAL1 in Neurosignaling Synaptic Plasticity and Cognitive Functions

Neurosigning and synaptic plasticity determine all major brain functions, cognitive abilities, and behavior. BMAL1 is localized in the pyramidal neurons of the hippocampal CA1 region, where it regulates synaptic plasticity in a circadian manner through interaction with CaMKIIa [61]. In the cerebral cortex, this transcription factor regulates the rhythmic expression of proteins involved in synapse structure formation (PLXND1 and CAMKV) in the mouse neocortex [57]. In this case, BMAL1 disruption in neurons affects cognitive functions (Table A1). For example, BMAL1-KO mice have decreased short-term memory and the ability to recognize a new social object, but only at a certain time (2 h after dawn), which indicates the role of circadian rhythms in cognitive functions [62]. BMAL1-KO in forebrain excitatory neurons leads to deficits in both acquisition and recall in the Barnes maze and abrogated time-of-day-dependent novel object location memory [63]. BMAL1-KO in mice leads to a decrease in hippocampal long-term potentiation and memory consolidation, which is expressed as impaired spatial and fear memory. These deficits are accompanied by a decrease in MAPK and cAMP signaling and a loss of their daily fluctuations in the hippocampus [64]. These data highlight the important role of the circadian factor BMAL1 in cognitive behavior and the necessity of taking into account the time of day in these studies with rodents.

BMAL1 may be involved in synaptic plasticity not only because of its activity in nerve cells but also via its activity in microglia and astrocytes [47,50]. Microglia regulate synaptic circuits by reducing the number of nonfunctional synapses [2]. Microglial phagocytosis can be caused by neighboring neurons under metabolic stress or during memory formation. Therefore, microglia provide a healthier microenvironment for neurons in an adaptive response setting [65]. Microglia-specific *Bmal1* knockdown (KD) improves cognitive function in mice fed a high-fat diet, accompanied by increased hippocampal formation of mature synaptic spines during learning, which is associated with increased microglial phagocytic capacity in the hippocampal radial layer (stratum radiatum). Additionally, there is some sexual dimorphism in this function [65]. On the other hand, in cultures of LPS-stimulated microglial cells (BMAL1 increased), phagocytic activity is also increased, and proteins that regulate synaptic plasticity (triggering receptor expressed on myeloid cells 1 (TREM1), BDNF, Copine-6, and synaptotagmin 1) are decreased, whereas *Bmal1* silencing reverses these changes [47]. Thus, there is crosstalk between inflammation and cognitive function, where BMAL1 plays an important role. Interestingly, daily blue light exposure (10 min per day) to specific-pathogen-free (SPF) mice during 6 months reduces the expression of circadian genes, including *Bmal1*, leads to memory and learning impairment, and accompanies the development of a proinflammatory microglial phenotype and an increase in the number of hippocampal macrophages, which stimulates neurodegeneration [66]. This may indicate a possible mechanism of degenerative processes that connects the microglial phenotype, circadian rhythm, and microbiome [67].

In aged mice, microglial BMAL1-KO suppresses long-term potentiation in the CA1 hippocampal region via a decrease in the level of the GluA1 subunit of the AMPA receptor, which is responsible for anchoring this receptor to the synapse membrane, and lacking synaptic opsonin C1q, which promotes microglia-induced synapse removal and a reduction in microglial lysosomal proteins (cluster of differentiation 68 (CD68), lysosomal associated membrane protein 1 (LAMP1), and p62). As a result, increased levels of pre- and postsynaptic proteins (synaptosome-associated protein 25 (SNAP25) and postsynaptic density 95 (PSD95), respectively) raised synaptic density due to immature synapse preservation and disruption of hippocampus-dependent memory, particularly a lack of new object recognition and spatial memory. Notably, dysfunction increases with age and is associated with age-related neurodegeneration, which indicates the importance of BMAL1 in the aging process and senile dysfunction of synaptic transmission [2].

Astrocytes are the part of the “threepartite synapse” where they regulate synaptic functions, their integrity, and neurotransmitter recycling [50]. BMAL1 may regulate astrocyte morphology by binding to the E-box element on the promoter of the Cortactin (Cttn) gene, which encodes an actin-binding protein. In young (6–8-week-old) BMAL1-KO mice, disruption of this process leads to cognitive impairment via dysregulation of synaptic neurotransmission [50]. Thus, BMAL1 in a wide variety of CNS cells ensures the correct functioning of synapses and neuronal signaling, thereby contributing to the cognitive functions of the brain.

No less important is the ability of BMAL1 to regulate neuronal signaling via neuromediators. Even BMAL1 haplodeficiency (BMAL1^+/−^) in mice disrupts the formation of fear memory due to neurotransmitter system changes in the PFC, such as increased levels of serotonin (5-HT) and decreased levels of dopamine (DA), gamma aminobutyric acid (GABA), Glu, and norepinephrine (NA). In addition, the expression of SIRT1. 5-HT2C receptor and dopamine transporter (DAT) levels are increased, whereas the expression of the enzymes indoleamine-2,3-dioxygenase (IDO), tryptophan hydroxylase 2 (TRH2), and tyrosine hydroxylase (TH) is reduced. All of these effects are accompanied by neuroinflammation and mitochondrial dysfunction, mostly due to disruption of the 5-HT pathway [68]. Induced transgenic dominant-negative BMAL1 expression (dnBMAL1) in the mouse forebrain or hippocampus leads to a defect in memory retrieval, which is associated with the suppression of DA-cAMP signaling in the hippocampus, including decreases in the levels of DA receptors (D1-R and D5-R) and GluA1-S845 phosphorylation (an essential starting signal for long-term stabilization of spatial memories) by protein kinase A (PKA) [69,70]. Additionally, astrocytic BMAL1 in mice may regulate GABA signaling and cognitive functions [71,72].

Among other factors, glucocorticoids play important roles in memory and cognitive functions [73]. BMAL1 plays a certain role in the function of the hypothalamic-pituitary-adrenal (HPA) axis. This protein is directly involved in the modulation of the sensitivity of GRE (glucocorticoid responsive element) promoters to glucocorticoid receptors through physical binding, preventing glucocorticoid-mediated gene transcription [74]. BMAL1 in the SCN is involved in controlling the circadian rhythm of corticosterone [75,76]. Moreover, BMAL1 in the adrenal gland regulates the response to adrenocorticotropic hormone (ACTH) by controlling genes implicated in cholesterol transport [77].

Thus, BMAL1 in most CNS cell types, besides the neurons, is directly or indirectly involved in the regulation of synaptic function and neurosignaling by controlling the circadian rhythm of intracellular cascades, neurotransmitter metabolism, and synapse maturation.

Summarizing all of the above, BMAL1 is an important protein for maintaining control of processes occurring in all cells of the nervous system (Table 2).

Dysfunction of BMAL1 in any type of CNS cell can alter the local microenvironment and impair neighboring cell function. Consequently, mutations in this gene are a predictor of nervous system diseases. Studies have identified *BMAL1* gene polymorphisms and mutations associated with human CNS diseases. To reveal in detail the mechanisms of BMAL1 in key pathological processes, many animal models with insufficient or redundant BMAL1 protein have been created, which we will discuss further (Table A1).

## 4. BMAL1 in CNS Pathologies

### 4.1. BMAL1 in CNS Organic Lesions

CNS organic lesions are accompanied by neuroinflammation and oxidative stress, which are mediated by microglia (Table A1). Specifically, microglia can undergo pyroptosis, which, unlike conventional apoptosis, leads to the creation of transmembrane pores, cellular edema, rupture, and the release of numerous inflammatory cytokines. In vivo and in vitro models of spinal cord injury have shown that BMAL1 inhibits pyroptosis-related proteins (NLR-family pyrin domain-containing protein 3 (NLRP3), caspase-1, apoptosis-associated speck-like protein containing a CARD (ASC), and gasdermine-N) and suppresses the activating effect of the transcription factor NF-kB on MMP9 (matrix metalloproteinase-9) endopeptidase expression, suppressing secondary spinal cord injury [44]. In a rat model of postoperative brain injury (excision of the right frontal lobe), BMAL1 decreased not only in microglia but also in cortical neurons and astrocytes, reaching a minimum on the third day after injury, accompanied by increased lipid peroxidation, apoptosis, oxidative stress, and neuronal damage in the injured area. In rats, normal levels of BMAL1 returned on day 7 [78]. Traumatic brain injury (TBI) in rats also leads to a decrease in BMAL1 levels in the cortex, reaching a minimum at 48 h after injury, with simultaneous increases in p38-MAPK activity and inflammation, whereas the administration of recombinant BMAL1 reduces the expression of the proinflammatory cytokines IL-4 and TNFα, edema, the severity of somatosensory disorders, and nerve cell necrosis and normalizes behavior [79].

The appearance of stroke is time-of-day dependent and causes damage to brain tissue. Within 4.5 h after ischemic stroke onset, compared with those in the control group, the relative protein levels of SIRT1 and BMAL1 in the peripheral blood decreased, whereas the levels of oxidative stress and inflammation markers increased. Moreover, comparisons of BMAL1 and SIRT1 levels across four different time subgroups in patients (00:00–05:59, 06:00–11:59, 12:00–17:59, and 18:00–23:59) revealed that expression levels were lowest in patients with ischemic stroke onset at 6:00–11:59. These findings suggest that the decreased expression of SIRT1 and BMAL1 is associated with a greater incidence of ischemic stroke in the morning via a decreased anti-inflammatory and antioxidant defense system [80]. In mouse models of ischemic stroke (Table A1) created at four different time points, ischemia induced at midnight was characterized by increased expression of BMAL1, PER1, CLOCK circadian proteins, AKT, and ERK-1/2 cell survival kinases compared with ischemia induced at other time points. Moreover, these mice have less pronounced brain edema, neurological deficits, and apoptosis [81]. It is important to note that stroke appearance is also sex-dependent. For instance, female mice in a stroke model sustain significantly larger cortical damage than males, and even more extensive than observed in *Bmal1*^−/−^ females. Interestingly, *Bmal1*^−/−^ females have significantly higher estradiol levels, which probably provide a neuroprotective effect during the subacute phase [82]. In a rat model of intracerebral hemorrhage (Table A1), BMAL1 protein levels decreased in brain tissues. Genetic BMAL1 overexpression in these animals reduced brain edema, BBB damage, and neuronal death, resulting in improved neurological function. This protective effect was mediated by alleviating oxidative stress and inflammation through activation of the NRF2 signaling pathway [23]. On the other hand, BMAL1 deficiency in a mouse model of spinal cord injury is associated with preservation of OL at the epicenter of damage and improvement in the recovery of locomotor function on day 42 after injury. Additionally, there is less BBB damage, neuroinflammation, and bleeding during the first seven days after injury in these animals [83].

Thus, BMAL1 mostly has a protective role in CNS organic lesions and neurological disease by reducing inflammation and oxidative stress, but in white matter damage, which affects OL, BMAL1 deficiency also has a protective effect (Table A1); hence, future therapeutic strategies may depend on target cells.

### 4.2. Developmental Disorders and Myelinization Disturbances

As mentioned above, BMAL1 is necessary for proper CNS cell composition, defines NPC differentiation and migration, and also protects neurogenic niches from oxidative stress (Table 2) [42,56]. BMAL1 plays a role in the pathogenesis of neurodevelopmental diseases (Table A1) such as the tuberous sclerosis complex, which occurs due to mutations in the human *TSC1* or *TSC2* genes, which are critical inhibitors of the mTOR pathway. Clinically, this disease manifests as epilepsy, intellectual disability, autism, and sleep disorders. Mouse models with TSC2-CO exhibit increased BMAL1 and increased mTOR activity, whereas haploinsufficiency of BMAL1 normalizes the circadian rhythms of behavior in these mice [28].

In autism spectrum disorders (ASD) associated with impaired central nervous system development, patients experience changes in circadian rhythms, among other characteristic symptoms [84]. BMAL1 is associated with human sociability, and its missense mutation (BMAL1 p.G24E) is found in individuals with ASD [85,86]. BMAL1^+/−^ mice have increased mTOR activity in the brain (cerebellum and forebrain) and exhibit behavior similar to ASD: they emit abnormal ultrasonic vocalizations during mother separation, lack sociability and recognition of social novelty, exhibit stereotypical behavior, have impaired movement coordination, and experience anxiety [84]. Global BMAL1-KO in mice also leads to ASD-like behavior with an abnormal density of Purkinje cerebellar cells and an immature morphology of dendritic spines. Electrophysiological recordings revealed increased excitatory and inhibitory synaptic transmission and a decreased frequency of Purkinje cell firing. Differential expression of genes associated with ASD and ataxia (*Ntng2*, *Mfrp*, *Nr4a2*, *Thbs1*, *Atxn1*, and *Atxn3*), as well as impaired regulation of the mammalian rapamycin 1 complex (mTORC1) signaling pathway, was detected in the cerebellums of BMAL1-KO mice. Moreover, BMAL1-KO only in Purkinje cells was sufficient to reproduce ASD-like behavioral and cellular changes [87].

Disruption of circadian rhythms is associated with an increase in the incidence of demyelinating diseases, such as multiple sclerosis [88]. Myelin damage via injury, autoimmune glial cell activation, or insufficient OL functions hinders the speed and direction of signal transmission along the nerve fiber [58,59]. Since BMAL1 is important for OPC, BMAL1-KO, which is specific for this type of cell during neurodevelopment, leads to myelin thinning, dysregulation of cognitive and motor functions in mice, and sleep fragmentation, which is related to multiple sclerosis (Table A1). OPC-specific BMAL1-KO in adults does not change the density of these cells but worsens remyelination at demyelinated lesion sites due to changes in the morphology and migration of OPCs [59]. Astrocyte-specific BMAL1 deletion in a cuprizone-induced model disrupted OPC migration into the demyelinating lesion area [58]. In an autoimmune encephalomyelitis model (an animal model of human demyelinating diseases), BMAL1 and time of day regulate the accumulation and activation of various immune cells. The loss of myeloid BMAL1 or midday immunization in vitro model stimulates inflammation in the CNS through the expansion and infiltration of IL-1b-secreting monocytes, which leads to an increase in pathogenic T cells with the IL-17^+^/IFN-^γ+^ phenotype [88]. Therefore, BMAL1 deficiency in OPCs, astrocytes, or myeloid cells has a negative effect on myelination via different mechanisms under pathological conditions.

Thus, BMAL1 is involved in the pathogenesis of neurodevelopmental and demyelinating disorders (Table A1). Notably, BMAL1 deficiency affects Purkinje cerebellar cell physiology via the mTORC1 signaling pathway that underlies ASD-related deficits. In demyelinating diseases, while BMAL1 deficiency in myeloid cells affects inflammatory processes, its deficiency in OPCs or astrocytes disrupts OPC migration and remyelination at the site of injury.

### 4.3. Neurodegeneration

While BMAL1 is an important factor for cell protection, it also contributes to neurodegeneration (Table A1). This is evidenced by findings that shRNA-induced decrease in *Bmal1* expression (LV-shBMAL1) exacerbates H_2_O_2_-induced neuronal death in primary cell cultures. Also, more pronounced striatal neurodegeneration is observed in *Bmal1*^+/–^ mice treated with the mitochondrial inhibitor 3-nitroproprionic acid. BMAL1-KO causes degeneration of synaptic terminals and disruption of cortical functional connections and oxidative damage to neurons [41]. So, BMAL1 deficiency in the context of oxidative stress is a major risk factor of neurodegeneration.

On the other hand, patients with temporal lobe epilepsy without hippocampal sclerosis (characterized by severe neuronal loss and gliosis in one or more hippocampal regions) have higher levels of BMAL1 in the DG than patients with severe loss of neuronal cells in the CA1 and CA4 regions, 50–60% loss of DG granule cells, and their dispersion [89]. In the hippocampus, NMDA-induced excitotoxicity reduces BMAL1 levels in DG granule cells before apoptosis, and this decrease is a signal for NPC differentiation in SVZ [55]. Collectively, these data highlight the dual role of BMAL1 in neurodegeneration: its activity is crucial for defense against oxidative stress, while its reduction promotes adult neurogenesis.

Aging is accompanied by circadian rhythm violation and oxidative stress, which leads to a wide range of brain changes and can provoke neurodegenerative diseases associated with the accumulation of pathological proteins such as beta-amyloid (Aβ) hyperphosphorylated tau protein, and alpha-synuclein (αSyn) [2,90,91]. Patients with early stages of Parkinson’s disease (PD) exhibit arrhythmic expression of circadian genes, impaired blood levels of cortisol and melatonin, impaired sleep quality, and decreased *BMAL1* mRNA levels in whole blood white cells, which is associated with the severity of PD symptoms [46]. Global postnatal BMAL1-KO in mouse models of tauopathy or alpha-synucleinopathy suppresses the aggregation of both tau protein and αSyn, reduces microgliosis and related pathology, while astrocytic BMAL1-KO in vivo prevents αSyn and tau accumulation by increasing astrocyte activation and the expression of the *Bag3* gene (an activator of macroautophagy) and, as a result, the phagocytosis of pathological proteins [90]. Thus, BMAL1-mediated astrocyte function is implicated in the pathology of PD.

Disruption of the DA signaling pathway is a key factor in PD pathogenesis. Insufficiency of this neurotransmitter pathway is also characteristic of the rotenone model of PD, which leads to a decrease in *Bmal1* expression in the cerebral cortex [92]. BMAL1 inactivation in a mouse model of PD (treated with 1-methyl-4-phenyl-1,2,4,5-tetrahydropyridine or MPTP) exacerbated the pathological phenotype: it increased motor impairments, promoted the loss of DA neurons in the compact part of the substantia nigra, enhanced microglial activation, and upregulated the expression of proinflammatory cytokines and astrocyte markers in the striatum [46]. BMAL1-KO in mouse striatal medium spiny neurons has a pronounced effect on motor functions, which is associated with a decrease in signaling through D1 receptors [93]. Global postnatal, pan-neuronal, or TH-neuron–specific BMAL1-KO caused the autonomous loss of TH+ neurons in the compact part of the substantia nigra. This effect was not observed in light-induced circadian rhythm violation by astrocytic or microglial BMAL1 removal; moreover, αSyn administration to BMAL1-KO mice did not cause additional loss of TH neurons, indicating that loss of neuronal BMAL1 is a main factor of cell damage in the DA system [94].

Thus, BMAL1 insufficiency in PD models (Table A1) negatively affects DA signaling by decreasing the cell survival rate but may protect neurons from tau and αSyn plaques via phagocytosis.

Increased expression of the *BMAL1* gene and protein has been shown in the brains of patients with Alzheimer’s disease (AD) and in mouse models of this disease [95,96]. In the cerebral cortex of patients with AD, the protein levels of CLOCK and BMAL1 are elevated in astrocytes, which reduce GFAP-positive filaments and suppress aerobic glycolysis and lactate production via a reduction in hexokinase 1 and lactate dehydrogenase levels that promote cytotoxicity via the activation of caspase-3-dependent apoptosis in these cells [95]. Interestingly, the DNA methylation status of BMAL1 in the frontal cortex of AD patients is correlated with disease stages, cognitive ability, and other associated symptoms. Increased methylation of CpG2 is associated with higher stages of the disease, but methylation of CpG1, CpG2, and CpG4 is positively correlated with vocabulary. CpG2 site methylation is also positively correlated with the ability to make quick decisions and mind flexibility, whereas CpG3 and CpG5 are associated with nighttime wakefulness, and CpG4 is correlated with symptoms of depression in AD patients, indicating that fine epigenetic *BMAL1* gene regulation plays a critical role in AD pathogenesis [18].

A lack of BMAL1, CLOCK, or NPAS2 in mice causes severe age-dependent astrogliosis in the cerebral cortex and hippocampus [41]. Astrocyte-specific BMAL1-KO mice, which were crossed with models of pathological protein accumulation (Aß-APP/PS1-21 and APPNL-GF, a faster and slower model, respectively), presented increased activation of astrocytes around Aß plaques; however, this astrogliosis did not affect plaque accumulation or neuron dystrophy models (Table A1) [91]. Thus, the loss of astrocytic *Bmal1* is not sufficient to trigger astrocyte-dependent plaque removal processes in AD, whereas in PD models, BMAL1 loss in astrocytes reduces accumulation of pathological proteins, highlighting a differential role for BMAL1 in the pathogenesis of these two neurodegenerative diseases [90,91].

Astrocytes located in the hypothalamus directly perceive nutrients and hormones, integrating metabolic information and modulating neuron responses, and participate in optimizing energy resources, including through BMAL1 [97,98]. BMAL1-KO in astrocytes led to accelerated aging, increased food intake, body weight, and age-related development of insulin resistance in mice, all without any changes in brain reward systems. This dysregulation is a hallmark of metabolic syndrome and increases the risk of cognitive impairment and neurodegenerative diseases [99,100]. In a mouse model of type 2 diabetes mellitus induced by a combination of a high-fat diet and streptozotocin (a substance that is toxic to pancreatic beta cells), animals exhibited impaired glycolipid metabolism, a reduced index of preference for a new object, and a new Y-maze sleeve. These changes were accompanied by decreased hippocampal expression of synaptic plasticity proteins (BDNF, synapsin 1, and synaptotagmin 1), MT1B (melatonin receptor 1B), and BMAL1. Similar changes were observed in palmitate-stimulated cell culture, whereas the plasmid-induced BMAL1 overexpression reduced these abnormalities [100]. Thus, there is a noticeable relationship between the metabolic function of astrocytic BMAL1, its role in the activation of astrocytes and astrocytic apoptosis, and cognitive impairment, which is a predictor of neurodegeneration.

In summary, BMAL1 is implicated in neurodegenerative diseases through distinct mechanisms. In PD it promotes the survival of DA neurons by mitigating inflammation and oxidative stress. Additionally, BMAL1 in astrocytes regulates phagocytic activity of these cells, influencing the clearance of pathological proteins in PD. In AD, astrocytic BMAL1 is involved in modulating cellular metabolism in the brain and apoptotic pathways, which contributes to disease progression but does not affect Aβ accumulation. Moreover, BMAL1 regulates NPC differentiation that plays a part in the severity of neurodegeneration in temporal lobe epilepsy.

### 4.4. Mood and Addictive Disorders

Circadian rhythm disruption is a predictor of mood disorders and, simultaneously, a symptom. To date, three *BMAL1* single nucleotide polymorphisms (SNPs) have been associated with bipolar disorder and related symptoms in patients with this disease: two of them (rs1481892 and rs7107287) are associated with anxiety, and one SNP (rs1481892) is associated with hyperthymia [101]. Later, it was shown that the rs7107287 variant in patients was also associated with cyclothymia, depression symptoms, stress, and the negative effects of seasonality on people’s well-being during the year [102]. The SNP rs2278749 is correlated with weight gain and food intake as a result of seasonal changes and may be a predictor of seasonal affective disorder (SAD) when it is combined with polymorphisms of other circadian genes [103]. According to the British Biobank data for white British individuals (excluding other Europeans), the BMAL1 variant rs34862781-G is significantly associated with anhedonia, rs745752200-ATG is associated with neuroticism, rs12419833-T is associated with risk-taking behavior, and the rs5789783-T polymorphism occurs in individuals who have ever smoked. In people of African-Caribbean origin, there is only a connection between the rs141886574-T genotype and mood instability [104]. In the Novosibirsk population (Russia, western Siberia), people aged 25–64 years who are carriers of *BMAL1* CT+TT alleles and the T allele have a relatively high risk of anxiety, especially among women, whereas the C/C genotype is the most common genotype in the population [105]. To investigate the mechanism of how BMAL1 is implicated in mood and addictive disorders, various animal models were created (Table A1).

Several studies have shown that animal models of anxiety- or depression-like phenotypes are associated with BMAL1 changes in the hippocampus. A long-term variable photoperiod (LVP), which causes circadian disruption in mice, induces anxiety- and depression-like behaviors accompanied by increased nocturnal expression of *Bmal1* in the PFC and hippocampus. At the same time, the number of mature OLs decreases in the medial PFC and hippocampal CA1 zone, progressively worsening with prolonged LVP exposure [60]. Sleep deprivation significantly increases anxiety levels, impairs cognitive function in mice, reduces BMAL1 and BDNF expression, and enhances oxidative stress in the hippocampus, whereas the injection of human recombinant protein (rhBMAL1) normalizes these deviations [106]. In mice with a genetic model of depression (Disc1-Q31L), which also has an abnormal stress response, a decrease in BMAL1 is shown in the hippocampal CA1 region and the lateral habenula (LHb) [107].

Models with genetic BMAL1 disruption have elucidated the BMAL1-dependent mechanisms associated with the development of mood disorders. Young crab-eating macaques (*Macaca fascicularis*) with genetically induced BMAL1 deficiency exhibit abnormal regulation of beta and gamma oscillations during the day and night, as demonstrated by continuous 24 h electroencephalographic monitoring. The disruption of beta oscillations in these animals was associated with self-harm and delusion-like behavior [108]. BMAL1-KO in macaque also reduces sleep duration, increases nighttime activity, decreases hormone levels (melatonin, testosterone, and dehydroepiandrosterone), and disrupts the rhythmicity of their production. These changes are accompanied by increased expression of proinflammatory cytokines in the blood, systemic inflammation, anxiety- and depression-like behaviors (linked to elevated cortisol levels), as well as sensory processing impairments resembling schizophrenia-like behavior. All these behavioral impairments were exacerbated under constant light [109]. SAD typically manifests in late autumn and winter due to insufficient daylight. Under winter simulation conditions, female CBA/N mice, which have elevated melatonin levels, exhibited depression-like behavior accompanied by decreased *Bmal1* expression. Moreover, BMAL1-KO also induces a depression-like phenotype in CBA/N mice without environmental influence [110]. Thus, it is evident that BMAL1 deficiency induces affective disorder-like states via hormonal dysregulation that is accompanied by systemic inflammation. Furthermore, neuron-specific BMAL1-KO in the mouse cerebral cortex leads to depression-like behavior, accompanied by reduced NA levels in the cortex [111]. This indicates that BMAL1 deficiency also affects neurotransmitter systems in the pathogenesis of mood disorder.

BMAL1 deficiency may also induce pathological conditions via HPA axis disruption. SCN-specific *Bmal1* KD induces a depression-like phenotype in mice, increases body weight via disruption of corticosterone circadian rhythms, and disrupts stress-induced corticosterone production without altering the levels of orexin A, corticotrophin-releasing hormone, or glutamate decarboxylase [75,76]. BMAL1-KO mice exhibit anhedonia, low corticosterone levels, and an impaired adrenal response to ACTH, which results in a lack of behavioral response to acute and subchronic stress despite stable blood corticosterone levels before and after stress exposure, whereas the increase in ACTH concentration remains evident [77].

The hyporeactivity of the HPA axis increases the likelihood of engaging in risky behavior, including substance use, and affective disorders are often comorbid with various types of addiction [112,113]. In turn, substance use also contributes to the development of mood disorders [114]. The voluntary consumption of psychoactive substances is influenced by external and internal factors, including circadian clock genes. In male and female mice with conditional BMAL1-KO in nucleus accumbens (NAc) neurons, alcohol intake and preference increase [115,116]. The LHb, which contains semiautonomous circadian clocks, is a negative regulator of the mesolimbic DA system and involved in alcohol addiction. LHb-specific BMAL1-KO in males stimulates voluntary alcohol consumption, whereas in females, it has a more complex effect: it slightly reduces voluntary alcohol intake but significantly decreases the consumption of an aversive alcohol solution (with added quinine) and post-abstinence alcohol intake. Notably, habenular BMAL1-KO did not affect anxiety or depressive behavior, suggesting that the role of BMAL1 in alcohol consumption is independent of the affective state [117].

The role of BMAL1 in drug addiction has been elucidated in only a few studies. Was shown that chronic morphine administration in mice increased the phosphorylation of striatal proteins at Tyr residues but did not affect BMAL1 or CLOCK proteins [118]. Nevertheless, BMAL1-KO mice exhibited decreased sensitization, diminished rewarding effects of cocaine, and reduced drug-seeking behavior due to DA-system alterations, such as the loss of rhythmic *D2* gene expression in the PFC, reduced TH, and increased monoamine oxidase-B (MAO-B) in the striatum after acute cocaine exposure. Additionally, BMAL1-KO mice presented reduced GluA1 in the PFC following chronic cocaine use [62]. This finding demonstrates that while BMAL1 is crucial for reward system function, its disruption may negatively affect certain neural functions (e.g., cognition) while positively influencing others (e.g., reducing addiction to drugs).

In summary, animal studies have elucidated that BMAL1 is involved in the pathogenesis of mood disorders (mostly depression- and anxiety-like phenotypes) through dysregulation of hormonal axes, including the HPA, inflammation, and neurotransmitter systems. Furthermore, in the brain reward system, BMAL1, through the DA signaling modulates addictive behavior.

## 5. BMAL1 as a Therapeutic Target for CNS Disorders

Based on the above, BMAL1 is a promising therapeutic target for treating a wide range of nervous system pathologies. However, few studies have investigated the pharmacological modulation of this protein, making this topic a relevant and emerging area of research.

In patients with Parkinson’s disease in stages 1–3 according to the Hoehn and Yahr scale (system for describing the motor deficit in PD, first stage—unilateral deficit with minimal disability, third stage—bilateral disease, mild to moderate disability with impaired postural reflexes; the last, fifth stage is confinement to bed or wheelchair unless aided), melatonin was shown to increase BMAL1 expression without normalizing sleep [119]. Propofol, the most widely used intravenous anesthetic in clinical practice, has been employed in treating patients with refractory chronic primary insomnia and is safe and effective for long-term sleep quality improvement. In a sleep deprivation paradigm in 8–12-week-old rats, propofol administration improved cognitive function and sleep structure and normalized sleep duration. Propofol also increased hippocampal and, to a lesser extent, hypothalamic BMAL1 protein expression. Additionally, this treatment shifted microglial activity from the proinflammatory M1 phenotype to the anti-inflammatory M2 phenotype [120]. The neurogenic small molecule ISX-9 (an isoxazole compound) can sustain higher and more stable circadian oscillation amplitudes. It improves daily metabolic rhythms in middle-aged mice, sleep homeostasis, increased delta power during the day, and increased locomotor activity at night by stimulating CaMKIIδ-mediated phosphorylation of BMAL1 at residues S513/S515/S516 [27].

Moreover, plant-derived compounds may effectively regulate BMAL1 levels. Epigallocatechin-3-gallate (EGCG), a substance found in green tea, acts as an antioxidant and a potential BMAL1 agonist. Rats with TBI received intraperitoneal EGCG injections immediately after surgery, which increased cortical BMAL1 levels; reduced edema, apoptosis, and neuronal damage; and lowered toxic lipid peroxidation products [78]. In a 6-OHDA (6-hydroxydopamine)-induced Parkinson’s disease cell model, dihydroisotanshinone I, a compound derived from Danshen (*Salvia miltiorrhiza*), attenuated cell death, suppressed ROS and caspase-3 activity, and increased SIRT1 expression while reducing BMAL1 levels and apoptosis [121].

A small molecule, Core Circadian Modulator (CCM), that targets the cavity in the PAS-B domain of BMAL1 and alters the functions of BMAL1 as a transcription factor. CCM induces dose-dependent downregulation of inflammatory and phagocytic pathways in macrophages [122]. Given the role of inflammation in CNS disorders, BMAL1-targeting small molecules, such as CCM, represent a promising therapeutic approach for treating these diseases. Moreover, modulation of proteins involved in BMAL1 circadian regulation may also indirectly affect the molecular mechanisms of diseases involving BMAL1 [123]. For example, the REV-ERB agonist SR9009 in the SAMP8 mouse model of AD reversed cognitive dysfunction of an aged mouse and reduced Aβ1–40 and 1–42 levels in the cortex, which is consistent with improved cognitive function. Furthermore, SR9009 treatment led to increased hippocampal PSD-95, cortical synaptophysin expression, and the number of synapses, suggesting improvement in synaptic function [124].

Chronotherapy is a treatment approach that takes into account the circadian rhythms of the patient. These approaches have shown efficiency in several CNS disorders [125]. Chronotherapeutic strategies can be divided into two categories: clocks as targets and clocks as modulators [126]. Time-restricted feeding is an example of the first category. A recent study showed that restoring peripheral clock rhythmicity and synchrony by time-restricted feeding normalizes body weight and glucose metabolism in SCN-specific BMAL1-KO mice in constant darkness [127]. Another approach from this category is red light therapy. It was shown that in rats subjected to prolonged LED light, scheduled red-light therapy attenuated anxiety-like behavior and regulated *Per1* and *Bmal1* gene expression in basal ganglia [126].

In summary, BMAL1 represents a promising therapeutic target for CNS disorders. However, direct pharmacological strategies to modulate its activity remain underexplored, and there is a lack of data on non-pharmacological interventions, such as physical exercise, specifically in the context of BMAL1 modulation for neurological conditions. Nevertheless, exercise-induced *Bmal1* upregulation in aged mouse livers suggests the feasibility of non-pharmacological approaches to affect BMAL1-mediated pathological mechanisms [128].

## 6. Sex Differences in BMAL1 Functions

The pathogenetic mechanisms of neurological disorders, such as anxiety and depressive disorders, addictions, AD, PD, multiple sclerosis, epilepsy, and ASD, differ between males and females [129,130,131,132,133,134,135]. Overall circadian gene expression is sex-dimorphic and more sustained in females [136]. In rats, some sex differences were observed in the robustness of clock gene expression in different brain structures: females had fewer robust rhythms in the medial PFC, more robust rhythms in the hippocampus, and a greater mesor in the medial amygdala. Furthermore, females with a regular estrous cycle had attenuated aggregate rhythms in clock gene expression in the PFC compared to noncycling females. These findings suggest that gonadal hormones modulate the expression of the molecular clock [137].

Although data on sex differences in *Bmal1* expression are lacking, studies in Mexican volcano mice (*Neotomodon alstoni*) indicate specific variation in heart, liver, and hypothalamic BMAL1 protein between males and females [138]. Nevertheless, BMAL1 changes may have sex-specific influence on mammals. Microglia-specific BMAL1-KD in female mice fed a high-fat diet leads to greater levels of cellular apoptosis and/or more effective microglia phagocytosis in the arcuate nucleus than in male mice [65]. *Bmal1^−/−^* females in the stroke model had a more severe infarct core, increased astrogliosis, and a larger volume of densely packed microglia in the cortex at day seven after stroke than *Bmal1^−/−^* males, but all these differences vanished by day 14 post-stroke [82]. As mentioned above, BMAL1 is involved in voluntary alcohol consumption. In females only NAc-specific BMAL1-KO induces this behavior, whereas striatal KO suppresses alcohol consumption. In contrast, BMAL1-KO in both structures of male mice induces this behavior [115,116]. Also, LHb-specific BMAL1-KO in females has a more complex effect than in males [117].

In summary, BMAL1 deficiency in males and females under pathological conditions may differently affect the underlying mechanisms. Many studies indicate sex differences in the efficiency of pharmacological and other therapies for CNS disorders [139,140]. Therefore, further investigation into the sex differences in BMAL1-mediated mechanisms will facilitate the development of more effective therapies for patients of both sexes.

## 7. Conclusions

Functions of BMAL1 in the CNS go far beyond circadian rhythm regulation. In this review, we show that this protein is involved in the control of oxidative stress, determines the phenotype of microglia and their activation, participates in the regulation of astrocyte morphology and functions, synaptic transmission, and the creation of the necessary microenvironment for neuronal function, and is actively involved in neuronal and glial cell development in embryogenesis and adulthood. In each specific process and cell type in the CNS, BMAL1 may have a protective or detrimental effect. A decrease in BMAL1 expression is a signal for the differentiation of NPC and OPC, but more often it leads to neurodegenerative disorders, neurosignaling deficiency, cognitive decline, and various neurological and psychopathological disorders. Despite the small number of studies devoted to the therapeutic use of BMAL1, this protein is obviously a promising target for the treatment of CNS pathologies. It is extremely important to take into account the individual molecular and cellular mechanisms underlying these diseases in order to choose the right strategy for influencing BMAL1. Also, sex differences in BMAL1 activity remain unclear, but according to available data, it has an effect on disease pathogenesis and, most likely, on therapy efficiency. All of this opens another important area for research on this topic.

## Figures and Tables

**Table 1:** BMAL1 molecular regulators.

Regulator	Effect
**Transcription factors**	
DEC1/DEC2	Bind to BMAL1 and/or compete with CLOCK:BMAL1 for E-boxes. Provides additional stabilization of circadian rhythms [19].
NF-κB	Suppresses CLOCK: BMAL1 activity, competes with CRY1 and the coactivator CBP/p300 for TAD binding. Provides a connection between circadian rhythms and inflammation [20].
DCNP1	Interacts with BMAL1 on the E-box of *N-acetyltransferase* gene, blocks its BMAL1-mediated transcription. Affects melatonin synthesis and involved in the development of depression [21].
**Post-transcriptional modulators**	
MiR-142-3p/miR-155	Repression of *Bmal1* mRNA translation by binding to 3′ untranslated region [22,23]
Alternative splicing (BMAL1a variant)	Dimerizes with CLOCK or BMAL1b and blocks their transport to the nucleus because BMAL1a have no N-terminal signal of nuclear localization [24]
**Post-translational modulators**	
GSK-3	Ser17/Tyr21 phosphorylation stimulates ubiquitination and BMAL1 proteasomal degradation. Stabilizes REV-ERBα and promotes its translocation into the nucleus, suppressing transcription of the *Bmal1* gene. [25,26].
CaMKIIδ	Phosphorylation at Ser513/515/516 enhances BMAL1 activity in response to Ca^2+^ influx [27].
TSC1/TSC2 (Tuberous sclerosis 1/2)	Inhibit the mTOR pathway and enhance BMAL1 translation, ubiquitination, and degradation or transport to the nucleus [28].
S6K1 (ribosomal protein S6 kinase beta-1)	Phosphorylation of Ser42 residue attracts BMAL1 to the ribosome during protein synthesis, ensuring BMAL1function as a translational factor [29].
SIRT1	Providing circadian transcription of *Bmal1*. Stimulation of CLOCK-dependent acetylation of Lys537 in the BMAL1 protein and repression of CLOCK:BMAL1 complex activity. Deacetylation of BMAL1 in response to a change in NAD^+^ concentration, reducing its ability to bind the E-box. In the SCN, it activates the transcription of *Bmal1* and *Clock* through PGC-1α (peroxisome proliferator-activated receptor gamma coactivator 1α). [30,31,32].
**Other**	
Cyclophilins (peptide prolyl isomerases)	Cis-trans isomerization of TAD around the Trp-Pro imide bond [6].
E-box motif CA**CG**TG	Most preferred for binding to BMAL1, it is present in different amounts in different tissues [33].
Methylation of CpG sites on the BMAL1 promoter	Epigenetic gene silencing [18]
CLOCK:BMAL1 complex	Promotes chromatin opening through the acetyltransferase activity of CLOCK, the recruitment of some additional transcription factors, and the insertion of a histone variant into the H2AZ nucleosome, which enhances the transcriptional activity of the complex [34,35].
Dopamine 1 receptor (D1) agonists	Reduces *Bmal1* expression in the SCN both during the day and at night [36].

**Table 2:** Main physiological functions of BMAL1 in different CNS cell types.

CNS Cell Type	BMAL1 Functions	Reference
Neurons	BMAL1-NRF2 pathway increases resistance to oxidative stress. Involved in monoamines and other neurotransmitters metabolism. Involved mitochondrial functions via 5-HT signaling.	[41,68]
Hypothalamic neurons	Associated with basal *Nf-κb* and *Il-6* mRNA expression. Involved in memory retrieval, via DA-cAMP signaling, including decreases in the levels of DA receptors (D1-R and D5-R) and GluA1-S845 phosphorylation by PKA.	[43,69,70]
Pyramidal CA1 neurons	Regulates synaptic plasticity in a circadian manner through interaction with CaMKIIa. Rhythmicity of MAPK and cAMP signaling.	[61,64,66]
Cerebral cortex neuron	Regulates the rhythmic expression of proteins involved in synapse structure formation (PLXND1 and CAMKV) in the mouse neocortex.	[57]
NPC	Regulates the expression of genes involved in ROS detoxification, including *catalase*, and enzymes involved in reduction in reactive quinones and oxidized proteins such as *Aldh2* and *Nqo1.*	[41,42]
	Involved in NPC survival via SIRT1, 70 kDa peroxisomal membrane protein (PMP70), Waf1/CIP1 and oxidative stress regulation. Determines differentiation vector between astroglia and neuronal in adults via regulation of proteome composition and its rhythmicity in the neocortex and hippocampus. Involved in protein expression of ENTPD2, which is involved in extracellular signaling during neurogenesis.	[56,57]
	Promoting radial neuronal migration in the embryonic cortex and proliferation of axonal projections. Decrease in DG and subsequently in SVZ is a signal for NPC proliferation in adults, stimulate transformation of NPC into OPC, whereas lack of BMAL1 in forebrain neurons decrease NPC proliferation but accelerates NPC migration in the rostral migration stream from the SVZ to the olfactory bulb, indicating a region-specific role in CNS development.	[42,53,55,58]
OPC	Regulate expression of genes related to the proliferation, density, morphology and migration of these cells and also OL differentiation genes. Circadian-dependent suppression of BMAL1 synthesis via the Wnt pathway inhibitors SFRP1 and SFRP5 (Secreted frizzled-related protein) produced by astrocytes stimulates oligodendrogenesis in the SVZ.	[58,59,60]
Astrocytes	Partially involved in glutathione-S-transferase signaling, suppresses cell-autonomous astrocyte activation and the expression of inflammatory genes. Play a part in processes of astrocyte autophagia: endocytosis, lysosomal protein degradation, and the accumulation of LAMP1- and RAB7-positive organelles via a TFEB-independent mechanism. May regulate astrocyte morphology by binding to the E-box element on the promoter of the *Cortactin* (*Cttn*) gene, which encodes an actin-binding protein. GABA signaling and cognitive functions.	[50,51,52,71,72]
Myeloid cells	ROS detoxification via NRF2.	[40]
Microglia cell	Regulate balance between pro- and anti-inflammatory microglial phenotype via regulation of proinflammatory, anti-inflammatory and antioxidant genes expression, as well as NF-κB inflammatory pathway proteins (IκBα and p65), glucose metabolism, phagocytic activity.	[45,46,47]
Microglia cell in the hippocampus and the arcuate nucleus	Regulate phagocytic microglial activity and cellular apoptosis, affect lisosomal proteins (CD68, p62 and LAMP1), thereby involved in synaptic microenvironment creation. Regulate synaptic morphology and AMPA-signaling via microglia-dependent mechanism.	[2,65]
Pericytes	controls permeability and integrity via the regulation of PDGFRß.	[49]

## Data Availability

No new data were created or analyzed in this study. Data sharing is not applicable to this article.

## Abbreviations

The following abbreviations are used in this manuscript:

ACTH	Adrenocorticotropic Hormone
AD	Alzheimer’s Disease
ASD	Autism Spectrum Disorders
BBB	Blood–Brain Barrier
bHLH	Basic Helix-Loop-Helix
BMAL1	Brain and Muscle ARNT-Like1
CaMK	Calmodulin-Dependent Protein Kinase
CLOCK	Circadian Locomotor Output Cycles Kaput
CNS	Central Nervous System
CRY	Cryptochrome
D1/2	Dopamine 1/2 Receptor
DA	Dopamine
DG	Dentate Gyrus
Glu	Glutamate
HPA	Hypothalamic-Pituitary-Adrenal Axis
IL	Interleukin
KD	Knockdown
KO	Knockout
LHb	Lateral Habenula
LPS	Lipopolysaccharide
MAPK	Mitogen Activated Protein Kinase
mTOR	Mammalian Target of Rapamycin
NF-κB	Nuclear Factor Kappa-Light-Chain-Enhancer of Activated B Cells
NPAS	Neuronal PAS Domain
NPC	Neuronal Precursor Cells
NRF2	Nuclear factor erythroid 2-related factor 2
OL	Oligodendrocytes
OPC	Oligodendrocyte Precursor Cells
PAS	Per-ARNT-Sim
PD	Parkinson’s Disease
Per	Period
PFC	Prefrontal Cortex
ROS	Reactive Oxygen Species
SCN	Suprachiasmatic Nucleus
SIRT1	Silent Information Regulator 1
SNP	Single Nucleotide Polymorphism
SVZ	Subventricular Zone
TAD	Transactivation Domain
TH	Tyrosine Hydroxylase
TSC	Tuberous Sclerosis
αSyn	Alpha-Synuclein
Aβ	Beta-Amyloid
5-HT	Serotonin

## Appendix A

**Table A1:** Animal models with BMAL1 changing.

BMAL1 Changing	Model Information	Effects	Reference
Genetic overexpression of BMAL1	Human recombinant BMAL1 + Rat with traumatic brain injury	↓ IL-4 and TNFα ↓ cerebral edema, severity of somatosensory disorders, necrosis of nerve cells; normalizes behavior	[79]
	Lentiviral *Bmal1* overexpression and antagomir-155 treatments + Rat with intracerebral hemorrhage	↓ oxidative stress, inflammation, brain edema, BBB damage, neuron death, and neurological dysfunction; activation of the NRF2 pathway	[23]
	Human recombinant BMAL1 + Sleep deprivation in mice	↓ anxiety; ↑ cognitive functions; ↓ oxidative stress in hippocampus; ↑ BDNF	[106]
BMAL1 enhancement as a result of model creation	Mice with a stroke model created at midnight	↓ cerebral edema, neuralgic deficiency and apoptosis ↑ AKT and ERK-1/2	[81]
	Circadian misalignment in mice and on the OPC cell culture (Oli-Neu)	↓ AKT/mTOR signaling and expression of OL differentiation genes	[60]
	Circadian misalignment via long-term variable photoperiod in mice	Anxiety and depression-like behavior; ↓ mature OL in the medial prefrontal cortex and CA1 area of the hippocampus; ↓ AKT/mTOR signaling	
	LPS stimulated rat (*Bmal1* ↑ in hippocampus but ↓ in hyppothalamus)	Depression-like behaviors; hyperactivity of the hypothalamic-pituitary-adrenal axis; hyperactivation of astrocyte and microglia; ↑ peripheral and central abundance of TNF-α, IL-6, and C-reactive protein; ↓ BDNF, TREM-1 receptor, Copine6, and Synaptotagmin1 but ↑ TREM-2 Alteration in the fluctuation of temperature, serum concentration of melatonin and corticosterone; ↓ CLOCK, PER2, CRY2 in the hippocampus	[47]
Genetic global KO	Mice *GAG-Cre^ERT2^*+; *Bmal1f/f*; *P30*1S; (+tautopathy and α-synucleopathy)	Prevents aggregation of tau protein, reduces microglia and atrophy of nerve fibers	[90]
	Mice *Arntl^tm1Bra/tm1Bra^* + spinal cord injury	Preservation of OL in the epicenter of damage; ↑ restoration of locomotor function; ↓ BBB damage, neuroinflammation, and bleeding during the first 7 days after injury	[83]
	Female mice *Bmal1*^−/−^ + spinal cord injury	↓ Pyroptosis-related proteins (NLRP3, caspase-1, ASC protein, gasdermine-N) ↓ NF-kB activity and MMP9 expression ↓ secondary damage	[44]
	Crab-eating macaque with BMAL1-KO	Dysregulation of β- and γ-oscillations of the electroencephalogram day and night; self-harm and delusion-like behavior; ↑ nighttime activity; ↓ and disruption of the rhythmic production of melatonin, testosterone, cortisol and dehydroepiandrosterone, systemic inflammation, anxiety and depressive—like and schizophrenia-like behavior	[109]
	Mice *CAG*-*Cre^ERT2^*; *Bmal1^f/f^*	Cell-autonomous loss of TH+ neurons in the compact part of the substantia nigra	[94]
	Mice *Bmal1*^−/−^	↓ hippocampal NPC pools, their scattered distribution; ↑ NPC survival rate ↑ differentiation of NPC into cells of the astroglial lineage; ↑ oxidative stress proteins (SIRT1, PMP70 and Waf1/CIP1); accelerated aging of the neurogenic niche of the hippocampus	[56]
		violation of spatial memory and memory of fear ↓ long-term potentiation in the hippocampus and memory consolidation; ↓ MAPK and cAMP signaling and loss of its daily fluctuations	[64]
		Cognitive impairment ↑ astrocyte soma, oxidative stress; ↓ small astrocytic processes; ↓ *Cortactin*	[50]
		anhedonia; ↓ the level of corticosterone; ↓ transcription of cholesterol transport genes in the adrenal cortex	[77]
		↓ sensitization, incentive effects of cocaine; ↓ drug-seeking behaviors; absence of rhythmic expression of the D2 receptor gene in the prefrontal cortex, ↓ TH, and ↑MAO-B in the striatum after a single dose of cocaine; ↓ GluA1 in the prefrontal cortex after chronic cocaine use	[62]
	Female mice *Arntl^tm1Bra/tm1Bra^*	↑ plasma estradiol level ↑ Gonadotropin releasing hormone-immunoreactive fibers in both the preoptic area	[82]
	Female mice *Arntl^tm1Bra/tm1Bra^* + Photothrombosis	↓ brain damage and astrogliosis in at 14 day after stroke than in wild type females ↓ volume of packed microglia at 14 day after stroke in than in wild type; vanished sex differences	[82]
	Mice *Bmal1*^−/−^ + MPTP	↑ motor disfunction; ↑ loss of DA neurons in the compact part of the substantia nigra, ↓ DA signaling; activation of microglia, expression of proinflammatory cytokines and astrocytes in the striatum	[46]
	Female mice *CBA/N* + *Bmal1*^−/−^	Depressive-like behavior	[110]
	Astrocyte culture from *CAG*-*Cre^ERT2^*; *Bmal1^f/f^* mice	Cell-autonomous activation of astrocytes; ↑ expression of inflammatory genes; ↓ signaling of glutathione-S-transferase; neuronal death	[51]
KO in brain cells	Mice *NestinCre^+^*; *Bmal1^f/f^*	↑ proliferation and differentiation of cells in the SVZ	[55]
		↑ Oxidative stress, activation of astroglia and microglia, degeneration of synaptic endings, disruption of cortical functional connections	[41]
		pericyte dysfunction and hyper permeability of the BBB; ↓ regulation of PDGFRß	[49]
	Mice *Bmal1^fl/fl^* + stereotaxis administration of AAV2/5-CAG-CRE-EGFP to turn off genes in the NAc or LHb	↑ alcohol consumption and preferences (KO in the nucleus accumbens)	[115]
		↑ voluntary alcohol consumption in males; ↓ aversive alcohol solution consumption and alcohol consumption after abstinence in females	[117]
KO in neurons	Mice *CaMK2a-Cre*; *Bmal1f/f*	Cell-autonomous loss of TH+ neurons in the compact part of the substantia nigra	[94]
		↓ assimilation and memorization of spatial information depending on the time of day	[63]
	*Mice* *CaMK2a-Cre^T29−1Stl/J^*; *B6.129S4(Cg)-Arntl^tm1Weit/J^* (KO in the forebran); NPC culture from these mice	↓ proliferation and acceleration of NPC migration to the olfactory bulb; ↑reelin concentrations in olfactory bulbs, astrogliosis; isolated NCPs: ↓ catalase and ROS detoxification gene expression	[42]
	Mice *Emx1-Cre*^+^; *Bmal1^f/f^* (KO in the cortex)	The rhythmic proteomic composition of the neocortex and hippocampus is disrupted; ↑ GFAP and FABP7; ↑ PLXND1 and CaMKV; ↑ ENTPD2	[57]
		depressive-like behavior; ↓ NA in the cortex	[41]
	Mice *Gpr88*-*Cre*^+^; *Bmal1 ^-/f^* (KO in the spiny neurons of the striatum)	Motor disfunction ↓ DA-signaling through D1 receptors	[93]
	Mice *Bmal1^f/f^:L7-Cre*; *Bmal1^f/f^* (KO in Purkinje cells)	ASD-like behavior; abnormal density of Purkinje cerebellar cells and immature morphology of their dendritic spines; ↑ excitatory and inhibitory synaptic transmission, ↓ frequencies of Purkinje cells firing; the regulation of the mTORC1 signaling pathway is disrupted	[87]
	Cell culture mHypoA-BMAL1-WT/F and mHypoA-BMAL1-KO/F + palmitate	Increases expression *Il-6* Suppresses basal expression *Nf-κb*	[43]
KO in myeloid cells/microglia	Mice *Bmal1^LoxP/LoxP^* *Lys-MCre*	↓ NRF2; ↑ ROS and IL-1β	[41]
	Aged mice *Cd11b^cre^*; *Bmal^lox/lox^*	↓ hippocampus-dependent memory; In the CA1 region of the hippocampus: ↓ Long-term signal potentiation, ↓ GluA1, ↑ SNAP25 and PSD95, ↓ C1q, lysosomal microglial dysfunction ↑ synaptic density due to the preservation of immature synapses	[2]
	Mice *Bmal1^LoxP/LoxP^*; *Lyz2^Cre^*	Inflammation in the CNS: ↑ IL-1β- secreting monocytes, ↑ T cells with the IL-17^+^/IFN-γ^+^ phenotype	[88]
KO in myeloid cells/silencing	BV2 cells + LPS or palmitate	↑ phosphorilation of IκBα and p65, ↑ ROS ↓ IL-6 и TNF-α	[46,47]
		↓ *Il-1b* and *Nox2* ↑ *Gsr* and *Hmox1* ↑ glucose consumption and phagocytosis	[45]
Microglial haplodeficiency	Mice *Bmal1*^lox/^-*Cx3cr1*^CreER^ + The high-fat diet	↑ cognitive functions; ↑ formation of mature spines in the hippocampus during learning; ↑ phagocytic ability of microglia during learning in the hippocampus	[65]
KO in astrocytes	Mice *Gfap-cre*; *Bmal1^f/f^* + Cuprizone	↑ oligodendrogenesis and neurogenesis; disruption of OL transport from the SVZ to the lesion site	[58]
	Mice *Aldh1l1-Cre^ERT2+^*; *Bmal1f/f*	↑ endocytosis; lysosomal breakdown of proteins; ↑ autophagosome-like structures inside astrocytes	[52]
		Cell-autonomous loss of TH+ neurons in the compact part of the substantia nigra	[94]
	Mice *Aldh1l1-Cre^ERT2+^*; *Bmal1f/f*; *P301S+* (on background of tautophaty genetic model)	↓ αSyn and Tau-protein; ↑ activation of astrocytes; ↑ phagocytosis of pathological proteins	[90]
	Mice *Glast-Cre^ERT2^*; *Bmal1^f/f^*	Circadian rhythm and spatial memory disorders; ↓ GABA transporters in astrocytes of the cortex ↑ inhibition of learning and memory circuits	[71]
	Mice *Aldh1l1*-Cre^ERT2^; *Bmal1*^f/f^ + fast and slow accumulation model Aβ (*APP/PS1-21* and *APPNL-GF*)	Increased activation of astrocytes around Aß plaques, however, this astrogliosis does not affect plaque accumulation or neuron dystrophy in any of the models	[91]
KO in OPC	Mice *NG2:Cre*+; *Bmal1^f/f^*	Disrupts the expression of genes associated with circadian rhythms, proliferation, density, morphology, and migration; thinning of myelin, impaired cognitive and motor functions, fragmentation of sleep; ↓ remyelination in adulthood	[59]
Silencing	Mice with SCN-specific BMAL1-KD	Circadian rhythm disorders, including corticosterone; attenuated increase in corticosterone levels in response to stress; helplessness, behavioral despair, anxiety	[75]
	BV2 Cells + LPS + siRNA	↑ survival rate ↓ phagocytic activity Reverse LPS-stimulated protein changes	[47]
	Culture of cortical cells from mice E17 C57/BL6 + LV-shBMAL1 + H_2_O_2_	Spontaneous neurite degeneration and increased cell death	[41]
Haplodeficiency	Mice *Bmal1*^+/–^	↑ mTOR activity in the cerebellum and forebrain ASD-like behavior	[84]
		violation of the formation of fear memory; in the prefrontal cortex: ↑ 5-HT ↓ DA, GABA, Glu, NA ↑ SIRT1, 5-HT2C and DAT ↓ IDO, TPH2 and TH; neuroinflammation and mitochondrial dysfunction	[68]
	Mice *Bmal1*^+/–^ + 3-nitroproprionic acid	Severe damage to the striatum	[41]
	Mice *Bmal1^+^*^/−^; *Tsc2*^+^^/−^	Normalization of the circadian rhythm of behavior	[28]
Model induced decrease in BMAL1	Mice + high-fat diet + streptozocin	↓ preferences of the new object and the Y-maze sleeve; ↓ BDNF, synapsin1, synaptotagmin1, MT1B in the hippocampus	[100]
	Mice SPF + blue light irradiation (10 min/day during 6 months)	Formation of the proinflammatory phenotype of microglia; ↑ hippocampal macrophages; ↑ neurodegeneration and memory impairment	[66]
	Rats with postoperative brain damage	↑ lipid peroxidation, apoptosis; neuronal disorders in the area of injury	[78]
	HT-22 cells + palmitate	Deficit in lipid metabolism ↓ proteins of synaptic plasticity	[100]
